# Surveillance of antenatal influenza vaccination: validity of current systems and recommendations for improvement

**DOI:** 10.1186/s12889-015-2234-z

**Published:** 2015-11-23

**Authors:** Annette K. Regan, Donna B. Mak, Hannah C. Moore, Lauren Tracey, Richard Saker, Catherine Jones, Paul V. Effler

**Affiliations:** School of Pathology and Laboratory Medicine, University of Western Australia, Crawley, Western Australia Australia; Western Australia Department of Health, Communicable Disease Control Directorate, Shenton Park, WA 6008 Australia; Wesfarmers Centre for Vaccines and Infectious Diseases, Telethon Kids Institute, University of Western Australia, Subiaco, Western Australia Australia; Joondalup Health Campus, Joondalup, Western Australia Australia; Infection Control, King Edward Memorial Hospital, Subiaco, Western Australia Australia

**Keywords:** Antenatal influenza vaccination, Influenza, Pregnancy, Surveillance. Vaccines, Immunisation

## Abstract

**Background:**

Although influenza vaccination is recommended during pregnancy as standard of care, limited surveillance data are available for monitoring uptake. Our aim was to evaluate the validity of existing surveillance in Western Australia for measuring antenatal influenza immunisations.

**Methods:**

The self-reported vaccination status of 563 women who delivered between April and October 2013 was compared against three passive data collection sources: a state-wide antenatal influenza vaccination database maintained by the Department of Health, a public maternity hospital database, and a private health service database. Sensitivity, specificity, and positive and negative predictive values were calculated for each system using self-report as the “gold standard.”

**Results:**

The state-wide antenatal vaccination database detected 45.7 % (95 % CI: 40.1–51.4 %) of influenza vaccinations, the public maternity hospital database detected 66.7 % (95 % CI: 55.1–76.9 %), and the private health service database detected 29.1 % (95 % CI: 20.5–39.4 %). Specificity exceeded 90 % and positive predictive values exceeded 80 % for each system. Sensitivity was lowest for women whose antenatal care was provided by a private obstetrician.

**Conclusions:**

Existing resources for surveillance of antenatal influenza vaccinations detect 29–67 % of vaccinations. Considering the importance of influenza immunisation as a public health intervention, particularly in pregnant women, improvements to routine monitoring of influenza vaccination is warranted.

## Background

To minimise the incidence of influenza infection in pregnant women and infants aged less than 6 months, the World Health Organization has recommended pregnant women be given the highest priority in seasonal influenza vaccination programs [1]. Due to the health benefits to both mother and infant, influenza vaccination has been recommended in Australia since 2009 [2]. Despite strong recommendations promoting vaccination, there is currently no comprehensive, population-based surveillance system for antenatal influenza vaccination nationally or in any Australian jurisdiction. Similar to surveillance systems in the United States [3] and other countries, estimates of influenza vaccine uptake in Australia typically rely on self-reported data collected by telephone survey. However, these surveys are not conducted routinely and often rely on a small sample of pregnant women. The most recent national estimate of antenatal influenza vaccine uptake in Australia (12.7 %) is based on results from a computer-assisted telephone survey conducted in 2009 of just over 10,000 Australian adults, 182 of whom were pregnant [4]. More recent publications have reported uptake within a single jurisdiction or health service, and are not based on routinely collected surveillance data [5–7].

In the absence of a surveillance system for monitoring antenatal vaccinations, WA Health has conducted an annual survey of pregnant women who delivered during influenza season to estimate the proportion of women who were vaccinated since 2012 [5]. While these surveys have been useful in tracking vaccine uptake in pregnant women and have demonstrated improvement from 10 % in 2009 to 36 % in 2013 [7], implementing annual surveys is resource-intensive and more efficient vaccine surveillance methods may be available for this population.

Other potential data sources for monitoring influenza vaccine uptake during pregnancy are summarised in Table 1. In 2012, WA Health established the Western Australian Influenza Vaccination Database (WAAIVD), a state-wide database of government-funded antenatal influenza vaccinations reported to WA Health, as provided by vaccination providers. Antenatal clinics, general practitioners, and hospitals submit reports which include the full name, date of birth, trimester of pregnancy, batch number and brand of influenza vaccine administered. Reports are generally submitted 1–2 days post-vaccination. Between March 2012 and September 2014, 11,427 doses of trivalent influenza vaccine were reported to WAAIVD. In addition to this database, some public and private maternity hospitals maintain electronic databases into which influenza vaccination status is entered after delivery and before hospital discharge. The accuracy and completeness of antenatal influenza vaccination status recorded in these systems is currently unknown.

**Table 1:** Sources of antenatal influenza vaccination information evaluated

	Source of influenza vaccination information		
	Western Australian Antenatal Influenza Vaccination Database	Health service B database	Hospital A database
Population covered	All pregnant women within Western Australia	Women who deliver in a hospital within health service B	Women who deliver at hospital A
Data collected	Full name, date of birth, vaccination provider, date of vaccination, brand and batch number of vaccine, and trimester of vaccination.	Vaccination status (yes/no)	Vaccination status (yes/no)
Time data collected and entered into database	At time of vaccination	After delivery and before hospital discharge	After delivery and before hospital discharge
Person responsible for data collection and entry	Immunisation provider	Health professional attending birth	Health professional attending birth

This study investigates the sensitivity, specificity and positive and negative predictive values of antenatal influenza vaccination status as recorded in the WAAIVD and maternity hospital databases using self-reported vaccinated status from an annual survey as the “gold standard.”

## Methods

Western Australia has a population of 2.5 million people, representing 11 % of the total Australian population [8]. Approximately 30,000 babies are delivered each year in Western Australia. Hospital A is a large public antenatal hospital in the Perth metropolitan area which provides maternity care for 10 % of the metropolitan population as well as high risk pregnancies across the state. Health service B is a private health service located in the north metropolitan area, which manages three hospitals providing private maternity care for 20 % of the metropolitan area.

**Fig. 1:**
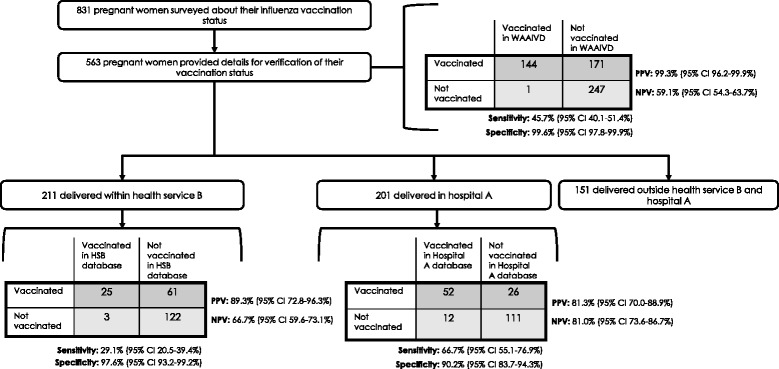
Assessment of antenatal influenza vaccination surveillance, Western Australia 2013. WAAIVD, Western Australia Antenatal Influenza Vaccination Database HSB, Health service B

In November and December 2013, 831 women who were pregnant during the 2013 influenza vaccination season and had given birth to a live baby between 07 April 2013 and 06 October 2013 were randomly selected from Western Australia’s statutory Midwives Notifications System. The Western Australia Department of Health conducted a 10-min computer-assisted telephone interview with selected women, which asked whether the woman had been vaccinated during her last pregnancy and reasons why or why she was not vaccinated [7]. Vaccinated women were asked for permission to verify their self-reported vaccination status with their immunisation provider; 563 women provided details for verification of their vaccination status with their immunisation provider: 211 delivered in health service B, 201 delivered in hospital A, and 151 delivered outside these services (Fig. 1). Because the WAAIVD is a state-wide database, the WAAIVD was screened for the vaccination status of all 563 women who provided details, based on full name and date of birth. Hospital A and health service B were provided the names and dates of birth of women who delivered within their respective health system and were asked to screen their electronic medical databases to confirm the women’s vaccination details. This project was reviewed and approved by the Western Australia Department of Health Human Research Ethics Committee.

We calculated the sensitivity, specificity, positive predictive values (PPVs), and negative predictive values (NPVs) for the WAAIVD and the electronic databases of participating maternity hospitals. A number of studies have shown that self-reported vaccination status is an accurate measure of vaccination status [9–11]. As a result, we decided to use self-reported antenatal influenza vaccination status as the “gold standard.” We calculated 95 % confidence intervals (CIs) using Wilson Score intervals.

Information on the woman’s age, antenatal care provider, chronic medical conditions, and postcode were available based on survey responses. The woman’s postcode was used to determine socioeconomic status, based on calculated socioeconomic indexes for areas (SEIFA) score, which is an indicator for socioeconomic conditions by postcode. SEIFA is comprised of several indices, the main index being the index of relative disadvantage which is derived from low income, low educational attainment, high unemployment and jobs in unskilled occupations [12]. Scores range from 700–1200. Estimates were stratified by quintile based on the distribution of scores in the state, with the lowest quintile indicating the woman was in the most disadvantaged socioeconomic group and the highest quintile indicating the woman was in the least disadvantaged socioeconomic group. Sensitivity and specificity were further stratified by subgroups of women for the WAAIVD, as there was sufficient sample size. Stratified sensitivity and specificity values were compared using z-tests at *α* = .05.

We also calculated the proportion of vaccinations which were recorded based on where the vaccine was administered and the trimester of pregnancy when the vaccine was administered. These proportions were compared across vaccination records using Pearson chi-square tests (*α* = .05).

## Results

The demographic characteristics of participating women are shown in Table 2. More than half of women were 30–39 years of age (57.4 %), were in the highest two socioeconomic quintiles (58.8 %), and had an undergraduate university degree or Training and Further Education (TAFE) qualification (52.2 %); 15.8 % had a chronic medical condition. Two in five women (44.1 %) received their antenatal care at a public antenatal hospital clinic; 37.3 % received care from a private obstetrician, 17.1 % from a general practitioner, and 1.4 % from another antenatal care provider.

**Table 2:** Demographic characteristics of study participants (*n* = 563), Western Australia 2013

	Number of women (%)	
Maternal age (in years)		
18–29	210	(37.3 %)
30–39	323	(57.4 %)
40–45	30	(5.3 %)
Residence		
Metropolitan	509	(90.4 %)
Rural/Remote	54	(9.6 %)
Socioeconomic status (SEIFA score)^a^		
Quintile 1 (Most disadvantaged)	13	(2.4 %)
Quintile 2	63	(11.5 %)
Quintile 3	150	(27.3 %)
Quintile 4	194	(35.3 %)
Quintile 5 (Least disadvantaged)	129	(23.5 %)
Highest level of education completed^b^		
≤High school graduate	175	(31.3 %)
Undergraduate/TAFE degree	292	(52.2 %)
Postgraduate degree	92	(16.5 %)
Existing Medical Conditions		
No chronic medical condition^c^	474	(84.2 %)
Has ≥1 chronic medical condition	89	(15.8 %)
Antenatal care provider^d^		
Public antenatal hospital clinic	247	(44.1 %)
General practitioner	96	(17.1 %)
Private obstetrician	209	(37.3 %)
Other provider^e^	8	(1.4 %)

^a^SEIFA, Socioeconomic indexes for areas; *n* = 549; 14 women had unknown SEIFA scores

^b^ *n* = 559; 4 women had unknown educational attainment

^c^Chronic medical conditions included asthma, chronic heart disease, diabetes, and chronic lung disorders

^d^ *n* = 560; 3 women did not provide details on their antenatal care provide

^e^Other antenatal care providers included independent midwives, community midwifery programs, and the Royal Australian Flying Doctors Services

A total of 37.9 % of women reported they had been vaccinated against influenza during their most recent pregnancy; 80.6 % of these could be verified with the immunisation provider. The majority of vaccinations were administered by a general practitioner (59.6 %); 20.3 % were administered by a public hospital antenatal clinic, and 16.8 % were administered by another immunisation provider, e.g. private hospital, community health clinic, and workplace immunisation clinic; 3.2 % of women did not specify who provided the vaccine. Most women reported being vaccinated in either their second (56.5 %) or third (31.7 %) trimester.

### Western Australia Antenatal Influenza Vaccination Database

A total of 45.7 % self-reported vaccinations were identified in the WAAIVD. The specificity and PPV of the WAAIVD was high (99.6 and 99.3 %, respectively). Table 3 displays the accuracy measures of WAAIVD by maternal age, SEIFA score, pre-existing medical conditions and antenatal care provider. Sensitivity did not significantly differ among women of varying ages, by socioeconomic status, or by the presence of existing clinical conditions. Sensitivity was significantly lower for women who received the majority of their antenatal care from a private obstetrician (33.6 % [95 % CI 26.2–41.8 %]) compared to women who received their care from a general practitioner (52.6 % [95 % CI 39.9–65.0 %]) or public hospital clinic (56.4 % [95 % CI 47.4–65.1 %], *p* < .01). A total of 84.4 % (95 % CI 73.6–91.3 %) of vaccinations administered at hospital A (a public maternity hospital) were captured by the WAAIVD; whereas, only 42.5 % (95 % CI 35.7–49.7 %) of vaccinations administered by a general practitioner and 13.2 % (95 % CI 6.5–24.8 %) administered by another immunisation provider were captured by the WAAIVD (*p* < .01) (data not shown). Significantly fewer vaccinations were recorded in the WAAIVD when they were administered in the first trimester (29.7 % [95 % CI 17.5–45.8 %]) than those administered in the third trimester (61.0 % [95 % CI 51.2–70.0 %]) (*p* < .01) (data not shown).

**Table 3:** Validity of the Western Australia Antenatal Influenza Vaccination Database (WAAIVD) for capturing antenatal vaccinations (*n* = 563), by patient characteristics

Subgroup	Recorded Vaccinations^a^	True Vaccinations^b^	Sensitivity	PPV^c^	Specificity	NPV^d^
	*n*	*N*	% (95 % CI)	% (95 % CI)	% (95 % CI)	% (95 % CI)
Total	145	144	45.7 (40.1–51.4)	99.3 (96.2–99.9)	99.6 (97.8–99.9)	59.1 (54.3–63.7)
Maternal age (years)						
18–29	49	107	44.9 (35.8–54.3)	97.9 (89.3–99.6)	99.0 (94.7–99.8)	63.3 (55.7–70.4)
30–39	85	187	45.5 (38.5–52.6)	100 (95.7–100)	100 (97.3–100)	57.1 (50.8–63.3)
40–45	11	21	52.4 (32.4–71.7)	100 (74.1–100)	100 (70.1–100)	47.4 (27.3–68.3)
Socioeconomic status (SEIFA score)^e^						
Quintile 1 (Most disadvantaged)	3	12	25.0 (5.5–57.2)	100 (43.9–100)	100 (56.6–100)	35.7 (16.3–61.2)
Quintile 2	17	32	53.1 (34.7–70.9)	100 (81.6–100)	100 (83.9–100)	57.1 (40.9–72.0)
Quintile 3	32	69	44.9 (32.9–57.4)	96.9 (84.3–99.4)	96.9 (83.8–99.4)	44.1 (32.9–55.9)
Quintile 4	31	56	55.4 (42.4–67.6)	100 (88.8–100)	100 (93.0–100)	67.1 (55.9–76.6)
Quintile 5 (Least disadvantaged)	56	138	40.6 (32.7–48.9)	100 (93.6–100)	100 (97.1–100)	61.1 (54.4–67.5)
Existing medical conditions						
Has ≥1 chronic medical condition	22	46	47.8 (34.1–61.9)	100 (85.1–100)	100 (91.8–100)	64.2 (52.2–74.6)
No chronic medical condition	123	269	45.3 (39.5–51.3)	99.2 (95.5–99.9)	99.5 (97.3–99.9)	58.1 (52.9–63.2)
Antenatal (AN) care provider^f^						
Public hospital AN clinic	67	117	56.4 (47.4–65.1)	98.5 (92.0–99.7)	99.2 (95.8–99.9)	71.7 (64.7–77.7)
General practice clinic	30	57	52.6 (39.9–65.0)	100 (88.6–100)	100 (91.0–100)	59.1 (47.0–70.1)
Private obstetrician	46	137	33.6 (26.2–41.8)^§§^	100 (92.3–100)	100 (94.9–100)	44.2 (36.8–51.8)^g^

^a^Recorded vaccinations were defined as vaccination events identified in the state’s antenatal influenza vaccination database, based on provider-reported vaccination information

^b^True vaccinations were defined as vaccination events self-reported by the woman during telephone interview

^c^PPV, positive predictive value

^d^NPV, negative predictive value

^e^SEIFA, Socioeconomic indexes for areas

^f^Antenatal care provider was defined as the healthcare professional who provided the majority of antenatal care as self-reported at the time of telephone interview

^g^Signifiant at *p* < .05

### Maternity hospital databases

After examining the electronic maternity hospital databases of hospital A and health service B, 47.0 % vaccinations were identified (Fig. 1). The sensitivity of hospital A’s system was significantly higher compared to that of health service B (66.7 and 29.1 %, respectively, *p* < .01). The specificity exceeded 90 % for each of the maternal hospital databases. However, specificity of hospital A’s maternity database was significantly lower (90.2 % [95 % CI 83.7–94.3 %]) compared to that of the WAAIVD (99.6 % [95 % CI 97.8–99.9 %], *p* < .05). The NPV of health service B’s database was significantly lower compared to NPV of hospital A’s database (66.7 and 81.0 %, respectively; *p* < .05). The majority of vaccinations administered at hospital A were recorded in a maternity hospital database (80.4 % [95 % CI 67.5–89.0 %]); whereas, 30.4 % (95 % CI 21.3–41.2 %) of vaccinations administered by a general practitioner were recorded, and 29.0 % (95 % CI 16.1–46.6 %) of vaccinations administered by some other immunisation provider were recorded (*p* < .01) (data not shown). No differences were observed by trimester of vaccination.

## Discussion

To our knowledge, this is the first formal evaluation of data sources for estimating antenatal influenza vaccinations using self-report as the “gold standard.” We found that systems which rely on provider-reported vaccination events or electronic medical records poorly capture influenza vaccinations administered to pregnant women, and the validity of these systems varies widely. A state-wide antenatal influenza vaccination database which relies on passive reporting from immunisation providers accurately recorded 46 % of influenza vaccinations administered to pregnant women. Electronic medical records within a public maternity hospital recorded 67 % of antenatal vaccinations, and public health service records recorded 29 %. These results indicate there is significant under-reporting of vaccinations administered to pregnant women in all systems.

The low sensitivity and negative predictive values identified in our study are perhaps not surprising, considering these systems rely on passive reporting from either immunisation providers or hospital staff. Although surveillance systems which rely on passive reporting have methodological advantages, such as low cost and relatively simple implementation, they often have low sensitivity and may not be representative. In the case of maternal vaccinations, it is apparent that immunisations are not comprehensively reported and entered, as demonstrated by our study. Previous research has demonstrated that incentives can be used to improve the timeliness and accuracy of recording vaccination events, and this may be one method for improving the validity of these systems [13, 14]. However, this introduces additional resource requirements for sustaining this surveillance activity. Education and targeted intervention may also improve recording; for example, in our setting, only one-third of vaccinations administered by a general practitioner were recorded in the state vaccination database. Targeted education could help improve provider reporting from these sites.

Vaccinations administered outside of traditional providers, such as places of employment, were also infrequently recorded in these systems, likely because these providers are unlikely to report vaccination events to the state government and are less often included in hospital records, as observed by our study. Previous investigations have shown that approximately 30 % of working-age adults receive influenza vaccines in non-traditional healthcare settings [15], and nine percent of vaccinated women in our study reported having been vaccinated at their workplace. Considering 13 % of immunisations provided outside of general practice or public hospital clinics were recorded by the WAAIVD and 29 % were recorded in the maternity hospital database, this is likely a large factor in the under-reporting of vaccinations to these systems and an area for improvement.

In the absence of a comprehensive system for monitoring adult vaccinations, such as an adult vaccination register, influenza vaccinations are difficult to routinely monitor in all adults. However, unique to antenatal vaccinations, there are two logical time points available for recording influenza vaccinations during pregnancy: once at vaccine administration, as the WAAIVD is structured, and again at delivery, as recorded by hospital databases. Data collection at the time of delivery can be logistically convenient as there are statutory requirements for the collection of information related to the pregnancy and birth [16]. Under the Health Act 1911, midwives are required to notify the Department of Health of the outcomes of all birth events within 28 h of birth by completing a Notification of Case Attended form [16]. Data collected in these forms are used to establish the Midwives Notification System, a state-based perinatal data collection [17]. The inclusion of influenza vaccination status in state or national perinatal data collections would establish an annual, electronic source of estimating influenza vaccine uptake in pregnant women. Other countries such as Canada choose to monitor influenza vaccination in this way. For example, the Nova Scotia Atlee Perinatal Database, a population perinatal database collects data on maternal health, details on the delivery, and information related to influenza vaccination [18]. Data are collected after hospital discharge and are based on standardised clinical forms and hospital records. These databases have proven useful for evaluating uptake and the effectiveness of maternal immunisation programs and could be used for similar purposes in Australia.

Globally, there are three types of systems which have been used to estimate influenza vaccine uptake during pregnancy: 1) population surveys; 2) healthcare utilisation, insurance claims, and pharmaceutical dispensary data; and 3) vaccination registries. Population surveys are a valid and reliable method for estimating uptake [5–7] and have been previously used for surveillance of maternal influenza immunisations in Australia [4]. However, they are time-consuming, resource intensive, and can be difficult to implement annually. Health service utilisation and health insurance claims databases have also been used for surveillance of maternal influenza immunisation. The UK General Practice Research Database, a primary care database containing de-identified health records from 8.4 % of the UK population, has been used to determine the proportion of women immunised against influenza [19, 20]. In the United States, patient-specific insurance claims data, such as Kaiser Permanente health plan membership data [21, 22] and LifeLink^TM^ Health Plan Claims Database [23] have been used to evaluate uptake of influenza vaccine during pregnancy. In France, a database of prescription use in the general population has been used to evaluate risks associated with drugs administered during pregnancy [24, 25]. While these databases tend to draw from a large population of unique members and have produced estimates similar to those of population surveys [18, 20], such databases are not necessarily designed to provide accurate vaccination uptake estimates. National databases or registries, such as the national database in Denmark which was established during the H1N1 pandemic to monitor H1N1 vaccination [26], are an ideal source of vaccination uptake data. However, the Denmark registry was restricted to pandemic vaccinations, and would need to be maintained annually for it to be useful in monitoring seasonal influenza vaccine uptake in pregnant women.

Electronic vaccination registries of the whole population, such as those of Denmark and a number of other countries in Europe [27, 28], could be used to collect seasonal influenza vaccination information needed to evaluate vaccination programs, in pregnant women and other target groups. Australia’s electronic immunisation registry is currently restricted to children under the age of seven years, and the case for a whole-of-life immunisation registry in Australia has been argued in the past [29, 30]. Expansion of this registry to adults would allow for monitoring of influenza vaccination during pregnancy as well as other target groups. Previous research has shown that electronic vaccination registries estimate vaccination status similar to self-reported vaccination status for women and high risk groups [31, 32], and electronic information is likely the most efficient source of routine vaccination information. Recent establishment of antenatal pertussis vaccination programs in the United Kingdom and the United States of America, and their imminent introduction in Australian jurisdictions underscore the importance of national surveillance systems for adult, including antenatal, vaccination uptake.

There are several limitations to consider when interpreting our data. First, we used self-reported vaccination status as the “gold standard” in this evaluation. While self-report has historically been proven to be a good measure of vaccination status, it could be argued that this is an imperfect measure. In our study, the majority of self-reported vaccinations could be verified by the immunisation provider (80.6 %); however, influenza vaccines administered in the workplace or by non-traditional providers could not be verified. Published literature support the validity of self-reported vaccination status in adults and indicate that false negative self-reports are extremely rare. The sensitivity of self-report has previously been shown to range from 90–100 % [9–11], and self-reported vaccination status is commonly used to estimate influenza vaccine coverage [33, 34]. However, given 20 % of self-reported records could not be verified, it is possible that some self-reported vaccinations were inaccurately classified as ‘true.’ Second, hospital A routinely offers influenza vaccination to its patients and reports these to WA Health. Hospital A is responsible for the majority of deliveries in public hospitals in Western Australia. It is likely that a large proportion of women who reported a public hospital clinic as their antenatal care provider were immunised at hospital A and were more likely to be recorded. Third, Hospital A and health service B provide maternity services within the metropolitan area of the state, and so the sample of women in this evaluation may not be representative of pregnant women across Western Australia. Finally, it is possible that a name and date of birth search in both databases was insufficient to identify women, which may lead to some discrepancies in the data.

## Conclusions

Monitoring influenza vaccine coverage is an integral component of national and state-based evaluation of immunisation programs, particularly for influenza, which is administered annually to a range of target groups [29, 33, 35]. Considering influenza vaccination is an important intervention for preventing disease in pregnant women and is a component of standard care for antenatal patients, surveillance of antenatal influenza vaccination could be improved. In addition to identifying contributing factors to the poor sensitivity of the systems evaluated in this study, additional systems for recording antenatal influenza vaccinations, such as recording vaccination status in perinatal data collections, are available which could be used to monitor this public health intervention. The gaps identified in this evaluation likely apply to other populations where monitoring vaccine uptake annually can be difficult. Exploration of alternative vaccination registers which include both children and adults, and record indication/s for vaccination, such as pregnancy and immunosuppression could potentially replace the fragmented immunisation registers currently available for specific age groups or vaccines. Such information is critical for providing data for monitoring coverage and evaluating disease prevention programs [29, 35].

## References

[1] World Health Organisation. Background paper on influenza vaccines and immunization. 2012. Accessed 15 October 2014. Available at: http://www.who.int/immunization/sage/meetings/2012/april/1_Background_Paper_Mar26_v13_cleaned.pdf.

[2] Department of Health and Ageing, Commonwealth of Australia (2013). Part 4: vaccine-preventable diseases - 4.7: influenza. The Australian immunisation handbook.

[3] Kennedy ED, Ahluwalia IB, Ding H, Lu PJ, Singleton JA, Bridges CB (2012). Monitoring seasonal influenza vaccination coverage in pregnant women in the United States. Am J Obstet Gynecol.

[4] Australian Institute of Health and Welfare, Commonwealth of Australia. 2009 Adult vaccination survey: summary results. March 2011. Available at: http://www.aihw.gov.au/WorkArea/DownloadAsset.aspx?id=10737418286.

[5] Taksdal SE, Mak DB, Joyce S, Tomlin S, Carcione D, Armstrong PK (2013). Predictors of uptake of influenza vaccination - a survey of pregnant women in Western Australia. Aust Fam Physician.

[6] Wiley KE, Massey PD, Cooper SC, Wood NJ, Ho J, Quinn HE (2013). Uptake of influenza vaccine by pregnant women: a cross-sectional survey. Med J Aust.

[7] Mak DB, Regan AK, Joyce S, Gibbs R, Effler PV. Antenatal care provider’s advice is the key determinant of influenza vaccination uptake in pregnant women. Aust N Z J Obstet Gynaecol. 2014, doi: 10.1111/ajo.12292. 10.1111/ajo.12292 25557858

[8] Australian Bureau of Statistics. Western Australia at a glance, 2014. Accessed 2 December 2014. Available at: http://www.abs.gov.au/ausstats/abs@.nsf/mf/1306.5.

[9] MacDonald R, Baken L, Nelson A, Nichol KL (1999). Validation of self-report of influenza and pneumococcal vaccination status in elderly outpatients. Am J Prev Med.

[10] Zimmerman RK, Raymund M, Janosky JE, Nowalk MP, Fine MJ (2003). Sensitivity and specificity of patient self-report of influenza and pneumococcal polysaccharide vaccinations among elderly outpatients in diverse patient care strata. Vaccine.

[11] Irving SA, Donahue JG, Shay DK, Ellis-Coyle TL, Belongia EA (2009). Evaluation of self-reported and registry-based influenza vaccination status in a Wisconsin cohort. Vaccine.

[12] Australian Bureau of Statistics. Socio-economic indexes for areas, 2011. November 2014. Accessed 2014 September 14. Available at: http://www.abs.gov.au/ausstats/abs@.nsf/mf/2033.0.55.001/.

[13] Fairbrother G, Siegel MJ, Friedman S, Kory PD, Butts GC (2001). Impact of financial incentives on documented immunization rates in the Inner City: results of a randomized controlled trial. Ambul Pediatr.

[14] Fairbrother G, Hanson KL, Friedman S, Butts GC (1999). The impact of physician bonuses, enhanced fees, and feedback on childhood immunization coverage rates. Am J Public Health.

[15] Singleton JA, Poel AJ, Lu PJ, Nichol KL, Iwane MK (2005). Where adults reported receiving influenza vaccination in the United States. Am J Infect Control.

[16] Western Australia Department of Health. Health (Notifications by Midwives) Regulations 1994. April 2014. Accessed 20 November 2014. Available at: http://www.slp.wa.gov.au/legislation/statutes.nsf/main_mrtitle_1563_homepage.html.

[17] Western Australia Department of Health. Midwives Notification System. November 2014. Accessed 20 November 2014. Available at: http://www.health.wa.gov.au/healthdata/statewide/midwives.cfm.

[18] Legge A, Dodds L, MacDonald NE, Scott J, McNeil S (2014). Rates and determinants of seasonal influenza vaccination in pregnancy and association with neonatal outcomes. CMAJ.

[19] Sammon CJ, Snowball J, McGrogan A, de Vries CS (2012). Evaluating the hazard of foetal death following H1N1 influenza vaccination: a population based cohort study in the UK GPRD. PLoS One.

[20] Sammon CJ, McGrogan A, Snowball J, de Vries CS (2013). Pandemic influenza vaccination during pregnancy: an investigation of vaccine uptake during the 2009/10 pandemic vaccination campaign in Great Britain. Hum Vaccin Immunother.

[21] Henninger M, Crane B, Naleway A (2013). Trends in influenza vaccine coverage in pregnant women, 2008 to 2012. Perm J.

[22] Naleway AL, Kurosky S, Henninger ML, Gold R, Nordin JD, Kharbanda EO (2014). Vaccinations given during pregnancy, 2002–2009: a descriptive study. Am J Prev Med.

[23] Toback SL, Beigi R, Tennis P, Sifakis F, Calingaert B, Ambrose CS (2011). Maternal outcomes among pregnant women receiving live attenuated influenza vaccine. Influenza Other Respir Viruses.

[24] Beau AB, Hurault-Delarue C, Vidal S, Guitard C, Vayssiere C, Petiot D (2014). Pandemic A/H1N1 influenza vaccination during pregnancy: a comparative study using the EFEMERIS database. Vaccine.

[25] Lacroix I, Hurault C, Sarramon MF, Guitard C, Berrebi A, Grau M (2009). Prescription of drugs during pregnancy: a study using EFEMERIS, the new French database. Eur J Clin Pharmacol.

[26] Pasternak B, Svanstrom H, Molgaard-Nielsen D, Krause TG, Emborg HD, Melbye M (2012). Vaccination against pandemic A/H1N1 2009 influenza in pregnancy and risk of fetal death: cohort study in Denmark. BMJ.

[27] Aguilar I, Reyes M, Martinez-Baz I, Guevara M, Albeniz E, Belza M, Castilla J. Use of the vaccination register to evaluate influenza vaccine coverage in seniors in the 2010/11 influenza season, Navarre, Spain. Euro Surveill. 2012;17(17):pii 20154. 10.2807/ese.17.17.20154-en 22551499

[28] Placzek H, Madoff LC (2011). The use of immunization registry-based data in vaccine effectiveness studies. Vaccine.

[29] Cheng AC, Hobbs CM, Robinson PM (2008). Australia needs an expanded immunisation register. Med J Aust.

[30] Public Health Association of Australia (2010). Resolutions PHAA 12th National immunisation conference.

[31] Jimenez-Garcia R, Hernandez-Barrera V, Rodriguez-Rieiro C, Carrasco Garrdo P, Lopez De Andres A, Jimenez-Trujillo I (2014). Comparison of self-report influenza vaccination coverage with data from a population based computerized vaccination registry and factors associated with discordance. Vaccine.

[32] Rolnick SJ, Parker ED, Nordin JD, Hedblom BD, Wei F, Kerby T (2013). Self-report compared to electronic medical record across eight adult vaccines: Do results vary by demographic factors?. Vaccine.

[33] Monto AS (2010). Seasonal influenza and vaccination coverage. Vaccine.

[34] Lu PJ, Santibanez TA, Williams WW, Zhang J, Ding H, Bryan L (2013). Surveillance of influenza vaccination coverage – United States, 2007–08 through 2001–12 influenza seasons. MMWR Surveill Summ.

[35] Centers for Disease Control and Prevention (2013). Prevention and control of seasonal influenza with vaccines recommendations of the advisory committee on immunization practices, United States, 2013–2014. MMWR.

